# Relationship between HER2 overexpression and long-term outcomes of early gastric cancer: a prospective observational study with a 6-year follow-up

**DOI:** 10.1186/s12876-022-02309-7

**Published:** 2022-05-13

**Authors:** Hui Li, Longsong Li, Nan Zhang, Zixin Wang, Ning Xu, Enqiang Linghu, Ningli Chai

**Affiliations:** 1 Department of Gastroenterology and Hepatology, First Medical Center of Chinese PLA General Hospital, Beijing, 100853 China; 2 Department of Gastroenterology, Air Force Medical Center, Beijing, 100142 China

**Keywords:** Human epidermal growth factor receptor 2, Overexpression, Early gastric cancer, Tumor recurrence, Mortality

## Abstract

**Background:**

Studies have confirmed the prognostic value of the expression status of human epidermal growth factor receptor 2 (HER2) in advanced gastric cancer. However, its role in early gastric cancer (EGC) remains largely unknown. This study explored the association between HER2 overexpression and clinical outcomes of patients with EGC.

**Methods:**

A total of 211 patients who had undergone endoscopic treatment for pN0 EGC were enrolled. The HER2 expression status was assessed using immunohistochemistry (IHC).

**Results:**

The prevalence of HER2 overexpression was 14.2%. HER2 overexpression showed a significant correlation with tumor location (*P* = 0.033). Multivariate analysis showed that HER2 overexpression was significantly associated with an increased risk of tumor recurrence in pN0 EGC (hazard ratio [HR] = 3.97; 95% confidence interval [CI] 1.30–12.14; *P* = 0.016) but not overall survival (OS) or disease-specific survival (DSS). Of the included patients, age was associated with OS (HR = 1.11; 95% CI 1.04–1.18; *P* = 0.002], whereas lymphovascular invasion was significantly associated with poor DSS (HR = 33.66; 95% CI 3.05–371.25; *P* = 0.004).

**Conclusion:**

This study shows that HER2 overexpression is significantly associated with tumor recurrence in pN0 EGC. Hence, Her2 testing at diagnosis is important and differential treatment and/or follow up strategies for patients with Her2 overexpression may merit future study.

**Supplementary Information:**

The online version contains supplementary material available at 10.1186/s12876-022-02309-7.

## Introduction

Gastric cancer remains an important cancer worldwide and imposes significant health-related and economic burden on the patients and society. According to the 2018 global cancer statistics, gastric cancer is the fifth most frequently diagnosed cancer and the third leading cause of cancer-related death globally [1]. With an estimated 0.46 million cases and 0.39 million associated deaths, gastric cancer is the third most frequent cancer and the second leading cause of cancer-related death in China [2]. Early gastric cancer (EGC) can be defined as mucosal carcinoma and/or submucosa-invasive carcinoma with or without lymph node metastasis (LNM). With the recent introduction of mass endoscopy screening and advancements in diagnostic technology, the detection rate of EGC has significantly increased in China [3]. Given similar efficacy with regard to the oncologic outcomes, practicing clinicians tend to administer endoscopic treatment over open or laparoscopic surgery. Evidence suggests that endoscopic resection (ER) yields similar long-term outcomes and considerable advantages regarding surgery time, hospital stay, costs, and complications as surgical resection; however, it is also associated with several disadvantages including higher risk of local recurrence and metachronous lesions [4, 5]. Although the long-term outcomes of patients with EGC receiving ER are excellent, several conventional risk factors, including depth of tumor invasion, lymph node status, and histologic type, are associated with poor prognosis [6–8]. Whether other unknown risk factors of EGC exist remains unclear.

Human epidermal growth factor receptor 2 (HER2) is a member of the HER family of tyrosine receptor kinases, encoded by proto-oncogene *ERBB2* on chromosome 17 [9]. Evidence suggests that HER2 can inhibit cell apoptosis and promote proliferation, thus essentially contributing to cancer cell survival and aggressiveness [10]. Recent studies have shown that HER2 expression is associated with the prognosis of advanced gastric cancer (AGC) [11]. The phase III ToGA trial established that trastuzumab, an anti-HER2 antibody, in combination with chemotherapy can be considered as a new standard treatment for patients with HER2-positive AGC [12]. However, whether HER2 is associated with the prognosis of EGC remains unclear. Therefore, this study explored whether the overexpression of HER2 is associated with poor prognosis of EGC.

## Methods

### Study population

Patients who were diagnosed with EGC between July 2009 and December 2013 by endoscopic screening and subsequent pathology at Chinese PLA General Hospital were included. The exclusion criteria were (1) other malignancies; (2) incomplete clinical or pathological data.

Patients were followed up by phone calls to determine survival status and cancer recurrence. The most recent follow-up was conducted in July 2020. All included patients were followed up for at least 6 years. Finally, 211 patients were included in the analysis. Additional file 1: Figure S1 shows the process of identification for participants. The study was conducted under the approval of the Ethics Board of the Chinese PLA General Hospital (ethics number: S2020-251-01), in accordance with the standards of the Declaration of Helsinki and Good Clinical Practice guidelines.

### Immunohistochemistry

HER2 expression status was determined using immunohistochemistry (IHC). Rabbit anti-human HER2 antibody was used (Roche Diagnostics Japan. Tokyo, Japan). Two pathologists individually scored the HER2 expression. Conflicts were resolved by a third pathologist’s interpretation. A semiquantitative approach, recommended by the National Comprehensive Cancer Network guidelines, was used to classify the expression status of HER2. We classified HER2 expression status into 2 groups: negative (0 and 1 +) and overexpression (2 + and 3 +) [13]. The definition for each was as follows: 0 = no membrane staining, or membrane responds to staining with < 10% of tumor cells; 1 +  = faint staining or barely recognizable staining in more than 10% of tumor cells; 2 +  = weak to moderate staining of the entire membrane in > 10% of tumor cells; 3 +  =  > 10% of tumor cells show a strong complete staining reaction in the entire membrane.

### Pathologic characteristics and definitions

Pathologic characteristics included depth of infiltration, lesion location, lesion morphology, lesion differentiation, tumor size, and lymphovascular infiltration. Depth of infiltration was categorized as mucosal or submucosal; lesion location as upper third, middle third, or lower third based on the lesion location on the longitudinal axis of the stomach; and lesion morphology as elevated (types 0-I, 0-IIa, 0-I + IIa, 0-IIa + IIb, 0-IIa + IIc), flat (type 0-IIb), or depressed (types 0-IIc, 0-III, 0-IIc + IIa, and 0-III + IIa) [14]. Histologically, tumors were characterized into 2 types: differentiated (papillary adenocarcinoma or moderately- and well-differentiated tubular adenocarcinoma) or undifferentiated (poorly differentiated tubular adenocarcinoma, signet-ring cell carcinoma, or mucinous adenocarcinoma). Tumor size was measured by the longest diameter of the lesion in the specimen and categorized into 2 groups: > 2 cm or ≤ 2 cm. Lymphovascular infiltration was classified into 2 groups: present or absent. In this study, three other clinical variables were also included: resection margin (positive or negative), current gastric ulcer (yes or no) and current *H. pylori* (*Hp*) infection when admitted to our hospital for EGC. Patients had previous *Hp* infection and have received successful *Hp* eradication therapy were classified as not having current *Hp* infection. Methodology of *Hp* diagnostic included serum antibody, rapid urease test, immunohistochemistry, ^13^C or ^14^C urea breath test, or stool antigen test.

### Prognostic outcomes

Prognostic outcomes of this study included overall survival (OS), disease-specific survival (DSS), and tumor recurrence. OS and DSS was ascertained by asking the family members the detailed information recorded on the death certificate of the patients.The OS period was determined from the date of endoscopic treatment for EGC until the date of death from any cause. The DSS period was determined from the date of endoscopic treatment for EGC to the date of death due to tumor recurrence (patients experienced recurrent GC, and died from dyscrasia or massive hemorrhage due to GC, or died from malignant event caused by GC distant metastasis, including severe lung infection, cerebral edema or hernia, or hepatic failure). The recurrence pattern in this study included both metachronous recurrence and metastatic recurrence. The tumor recurrence period was defined as the time from endoscopic treatment for EGC to the date of detection of new gastric cancer. Tumor recurrence was identified using routine abdomen-pelvis computed tomography and endoscopy after endoscopic treatment.

### Statistical analysis

We reported and compared the baseline clinicopathologic characteristics of included patients according to HER2 expression status, using the chi-square test or Fisher exact test for categorical data and the Student t test for continuous data. Multivariate Cox regression with stepwise backward selection was applied to identify factors associated with prognostic outcomes. For OS and DSS, 8 factors (age, sex, tumor size, depth of invasion, resection margin status, lymphovascular invasion, tumor differentiation, and HER2 expression status) were included in the model. For tumor recurrence, 2 additional factors (recent history of gastric ulcer and HP infection) were added in the model. These factors were chosen because they were considered potentially clinically relevant for each outcome. Survival curves for each prognostic outcome were generated using the Kaplan–Meier method and were compared using the log-rank test. Statistical analyses were performed using SPSS 26.0 for Windows (SPSS, Inc., Chicago, IL). Survival curves were graphed using GraphPad Prism 8 (GraphPad Prism version 8.1, GraphPad Software, La Jolla, CA). Two-tailed *P* values < 0.05 were considered statistically significant.

## Results

### Baseline characteristics of patients with EGC

A total of 211 patients who received endoscopic treatment for EGC were finally included. Baseline characteristics of included patients are summarized in Table 1. Of the 211 patients, 162 (76.8%) were men, and the mean age was 61.6 years (range 40–83 years). Of total, 30 patients (14.2%) had gastric ulcer, and 26 (12.3%) had a HP infection before endoscopic treatment. The most common lesion location was the lower third (116, 55.0%) on the longitudinal axis. The most common gross appearance of the lesion was of the elevated type (93, 44.1%). With regard to invasion depth, 193 patients (91.4%) had mucosal cancer. The differentiated-type EGC was significantly more prevalent than the undifferentiated-type EGC (n = 200, 94.8%), and 182 patients (86.3%) had a tumor size ≤ 2 cm. Furthermore, only 4 patients (1.9%) had lymphovascular infiltration. Finally, according to our definition, 30 patients (14.2%) had HER2 overexpression.

**Table 1:** Baseline characteristics of patients with EGC

Item	Total (n = 211)	%
*Age (years)*		
Average	61.6	
Range	40–83	
*Sex*		
Male	162	76.8
Female	49	23.2
*Tumor location*		
Upper 1/3	63	29.9
Middle 1/3	32	15.2
Lower 1/3	116	55.0
*Gross type*		
Elevated	93	44.1
Flat	71	33.6
Depressed	47	22.3
*Depth of invasion*		
Mucous layer	193	91.4
Submucosa	18	8.5
*Differentiated types*		
Differentiated	200	94.8
Undifferentiated	11	5.2
*Tumor size*		
≤ 2 cm	182	86.3
> 2 cm	29	13.7
*Lymphovascular infiltration*		
Present	4	1.9
Absent	207	98.1
*Resection margin*		
Positive	7	3.3
Negative	204	96.7
Current gastric ulcer	30	14.2
Current HP infection	26	12.3
HER2 overexpression	30	14.2

*EGC* early gastric cancer; *HP* *H. pylori*

### Clinicopathologic characteristics according to HER2 protein expression status

The clinicopathologic characteristics per HER2 protein expression status as assessed using IHC are summarized in Table 2. A significant correlation was observed between HER2 status and tumor location (*P* = 0.033) (Table 2). No significant associations were seen between HER2 expression and age, sex, history of gastric ulcer, HP infection, morphologic type, invasion depth, differentiated type EGC, proportions of large tumors (> 2 cm), and lymphovascular infiltration.

**Table 2:** Clinicopathological characteristics according to HER2 protein expression status

Item	HER2 negative N = 181	HER2 overexpression N = 30	*P* value
Age (year**,** Mean ± SD)	61.4 ± 9.7	62.3 ± 10.3	0.737
Sex (Male, %)	138 (76.2)	24 (80.0)	0.652
*Tumor location (n/%)a*			0.033
Upper 1/3	49 (27.1)	14 (46.7)	
Middle 1/3	26 (14.4)	6 (20.0)	
Lower 1/3	106 (58.6)	10 (33.3)	
*Gross type (n/%)*			0.183
Elevated	79 (43.6)	14 (46.7)	
Flat	58 (32.0)	13 (43.3)	
Depressed	44 (24.3)	3 (6.4)	
*Depth of invasion (n/%) *^*a*^			0.147
Mucous layer	168 (92.8)	25 (83.3)	
Submucosa	13 (7.2)	5 (16.7)	
*Differentiated types (n/%)*^*a*^			0.371
Differentiated	170 (93.9)	30 (100.0)	
Undifferentiated	11 (6.1)	0 (0.0)	
*Tumor size (n/%) a*			0.775
≤ 2 cm	155 (85.6)	27 (90.0)	
> 2 cm	26 (14.4)	3 (10.0)	
*Lymphovascular infiltration (n/%)*^*a*^			0.461
Present	3 (1.7)	1 (3.3)	
Absent	178 (98.3)	29 (96.7)	
*Resection margin (n/%)*^*a*^			1.000
Positive	6 (3.3)	1 (3.3)	
Negative	175 (96.7)	29 (96.7)	
Current gastric ulcer^a^	26 (14.4)	4 (13.3)	1.000
Current HP infection^a^	24 (13.3)	2 (6.7)	0.547

^a^Tested by Fisher exact test

### Association between HER2 expression and prognostic outcomes

**Fig. 1:**
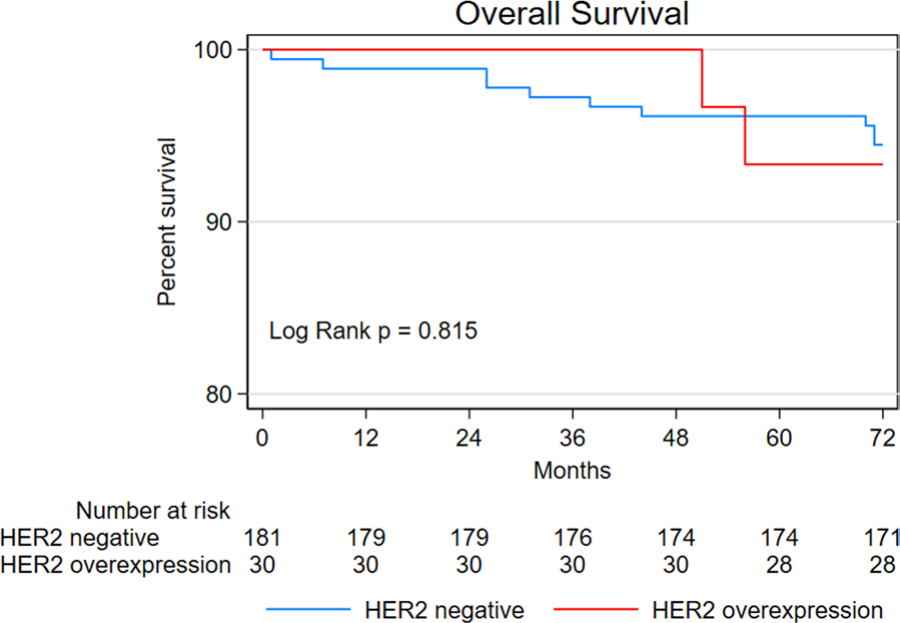
Kaplan–Meier curves for overall survival

**Fig. 2:**
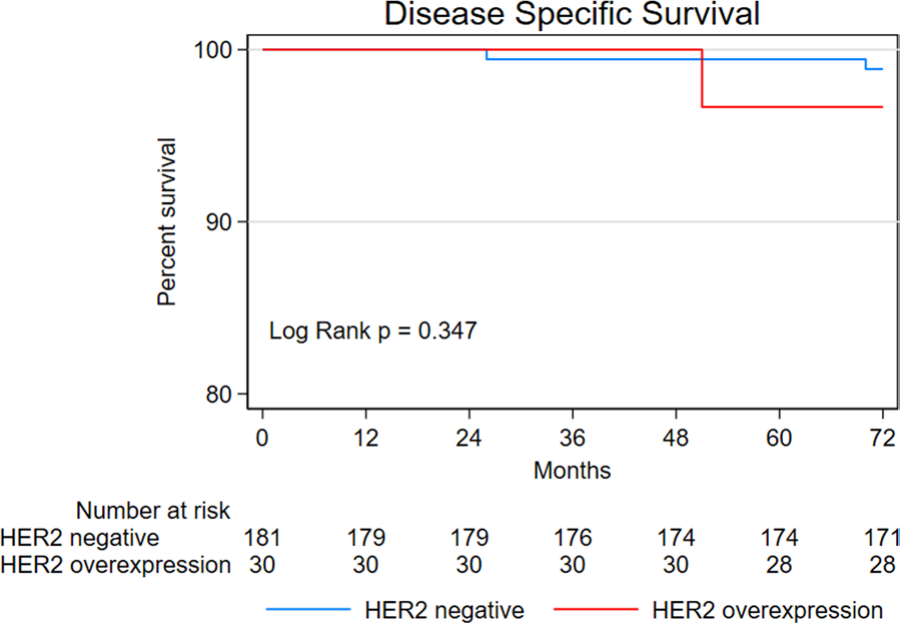
Kaplan–Meier curves for disease specific survival

**Fig. 3:**
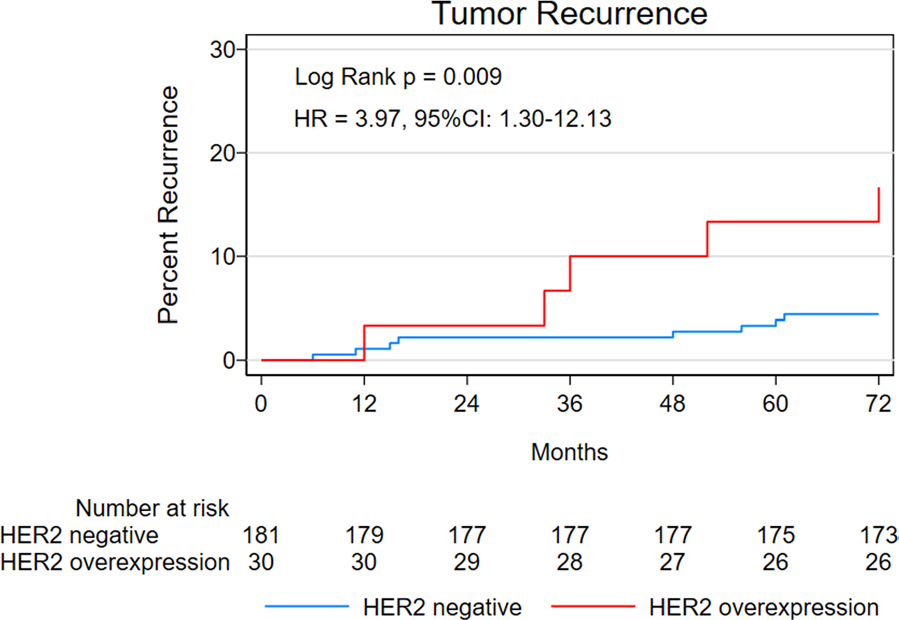
Kaplan–Meier curves for tumor recurrence

To examine the association between HER2 expression and prognostic outcomes, multivariate analyses were performed using the Cox proportional hazard model with backward elimination. The analyses revealed significant associations between age and poor OS (hazard ratio [HR] = 1.11; 95% confidence interval [CI] 1.04–1.18; *P* = 0.002), lymphovascular invasion and poor DSS (HR = 33.66; 95% CI 3.05–371.25; *P* = 0.004), and HER2 overexpression and tumor recurrence (HR = 3.97; 95% CI 1.30–12.14; *P* = 0.016)(Table 3). The log-rank test revealed a significant difference in tumor recurrence but not OS and DSS between HER2-negative patients and patients with HER2 overexpression (Figs. 1, 2, 3).

**Table 3:** Multivariate analyses for prognostic outcomes

Outcomes	*P*	Coefficient	SE	Wald	HR (95% CI)
*OS*					
Age (per year)	0.002	0.103	0.033	9.773	1.11 (1.04–1.18)
*DSS*					
Lymphovascular infiltration	0.004	3.516	1.225	8.243	33.66 (3.05–371.25)
*Tumor recurrence*					
HER2 overexpression	0.016	1.378	0.570	5.843	3.97 (1.30–12.14)

### Sensitivity analysis

A sensitivity analysis was conducted by excluding the patients lost to follow-up. Sensitivity analysis showed the similar results (Additional file 2: Table S1).

## Discussion

This study aimed to examine the prognostic utility of HER2 expression status in patients with EGC who received endoscopic treatment. We followed up 211 patients with EGC for a period of 72 months and found that HER2 overexpression was only associated with increased risk of tumor recurrence, not OS or DSS. These results are novel and would inform future studies.

Prevalence of HER2 positivity in patients with gastric cancer varies across studies. A review by Boku et al. showed that the prevalence ranged from 3.8 to 36.6% [15]. However, owing to the lack of studies, determining the true prevalence of HER2 positivity in patients with EGC is challenging. In this study, we found that the prevalence of HER2 overexpression is 14.2%, which is in line with that reported by Yan et al. [16]. In our study, the HER2 expression status was only associated with tumor location. However, Yan et al. and Han et al. both found that the HER2 positivity rate was associated with differentiated types, unlike in our study [16, 17]. This difference could be attributed to the inclusion of only those patients who received endoscopic treatment and a small proportion of patients with the undifferentiated type (5.7%) in our study. The studies by Yan et al. and Han et al. included patients who received surgical treatment, and the proportions of patients with the undifferentiated type were relatively high (35.8% and 53.5%, respectively) in their studies [16, 17]. Future studies are warranted to further explore the association between clinicopathologic characteristics and HER2 status.

Previous studies have shown that HER2 expression status is strongly associated with the prognosis of patients with AGC [11, 18]; however, only few studies have focused on the association between HER2 expression status and the clinical outcomes of patients with EGC. Yan et al. assessed the expression of HER2 in surgical specimens of 67 patients with EGC and without LNM who received surgical resection and investigated the association between HER2 expression status and OS [16]. They found that the prevalence of HER2 positivity was 16.4% and that HER2 positivity was associated with poor OS for EGC (HR = 1.384; 95.0% CI 1.142–1.897; *P* = 0.005) [16]. However, our results showed no association between HER2 overexpression and OS or DSS. These differences could likely be explained by the following reasons. First, Yan et al. included patients treated surgically, whereas we only included patients receiving endoscopic treatment. Second, the study by Yan et al. had a small sample size, which could have affected the reliability of the statistical estimation of the study outcome.

A significant result of our study is the finding that HER2 overexpression is correlated with a > fourfold higher risk of tumor recurrence in patients with EGC. The potential underlying mechanisms of this result are unclear. However, evidence suggests that HER2 immunoexpression is significantly associated with the development of micrometastases, which could lead to tumor recurrence [19]. In addition, Han et al. retrospectively reviewed 727 patients who underwent surgical treatment for EGC and evaluated the clinical significance of HER2 overexpression in these patients. Their results indicate that HER2 overexpression as a good predictive marker of LNM in patients with undifferentiated EGC (odds ratio, 6.49; 95% CI 1.12–37.37) [17]. LNM has long been confirmed as a significant risk factor for tumor recurrence. Moreover, previous studies have shown that HER2 overexpression is associated with recurrence or recurrence-free survival in breast cancer and gastric cancer [20–23]. These reports along with our results indicate that further studies are needed to clarify the clinical significance of HER2 overexpression in tumor recurrence in patients with EGC.

According to the ToGA trial, HER2 is a target in patients with AGC with HER2 overexpression [12]. An HER2 test is recommended when metastatic gastric adenocarcinoma is present or suspected. Overall, studies investigating the association between HER2 overexpression and prognosis of EGC are yet in the preliminary stages. However, these studies indicate that the importance of HER2 overexpression in EGC should not be overlooked. Implementing strict endoscopic follow-up after endoscopic treatment or surgical intervention rather than endoscopic treatment may be necessary given HER2 overexpression may play a significant role in EGC, as it does in AGC. We recommend performing an HER2 expression test in all patients with EGC.

Our study has several limitations. First, this was a single-center study. Second, statistical power might be insufficient because of the small sample size and outcome events. Third, many patients were lost to follow-up, which could have affected the results. Finally, fluorescence in situ hybridization (FISH) was not performed for IHC 2 + patients, which could have resulted in the misclassification of IHC 2 + and FISH ( −) patients into the HER2 overexpression group. However, in our study, 6 patients in the HER2 overexpression group were IHC 2 + . Hence, the effect of this misclassification was probably minimal.

## Conclusion

In conclusion, HER2 overexpression is an independent risk factor for tumor recurrence in patients with EGC who receive endoscopic treatment. Therefore, HER2 may have clinical significance in EGC as it does in AGC. Clinicians should hence be cautious of the possibility of tumor recurrence in patients with EGC who have HER2 overexpression. Further studies are warranted to confirm the prognostic role of HER2 expression in patients with EGC.

## Supplementary Information

**Additional file 1.** Flowchart of participants identification.

**Additional file 2.** Results of sensitivity analysis by excluding the patients lost to follow-up.

## Data Availability

The original contributions presented in the study are included in the article, further inquiries can be directed to the corresponding author.
