# Supplementary figures and images for: Relationship between HER2 overexpression and long-term outcomes of early gastric cancer: a prospective observational study with a 6-year follow-up
Source: BMC Gastroenterol. 2022 May 13;22:238. doi: 10.1186/s12876-022-02309-7 (PMC9102633; doi:10.1186/s12876-022-02309-7)

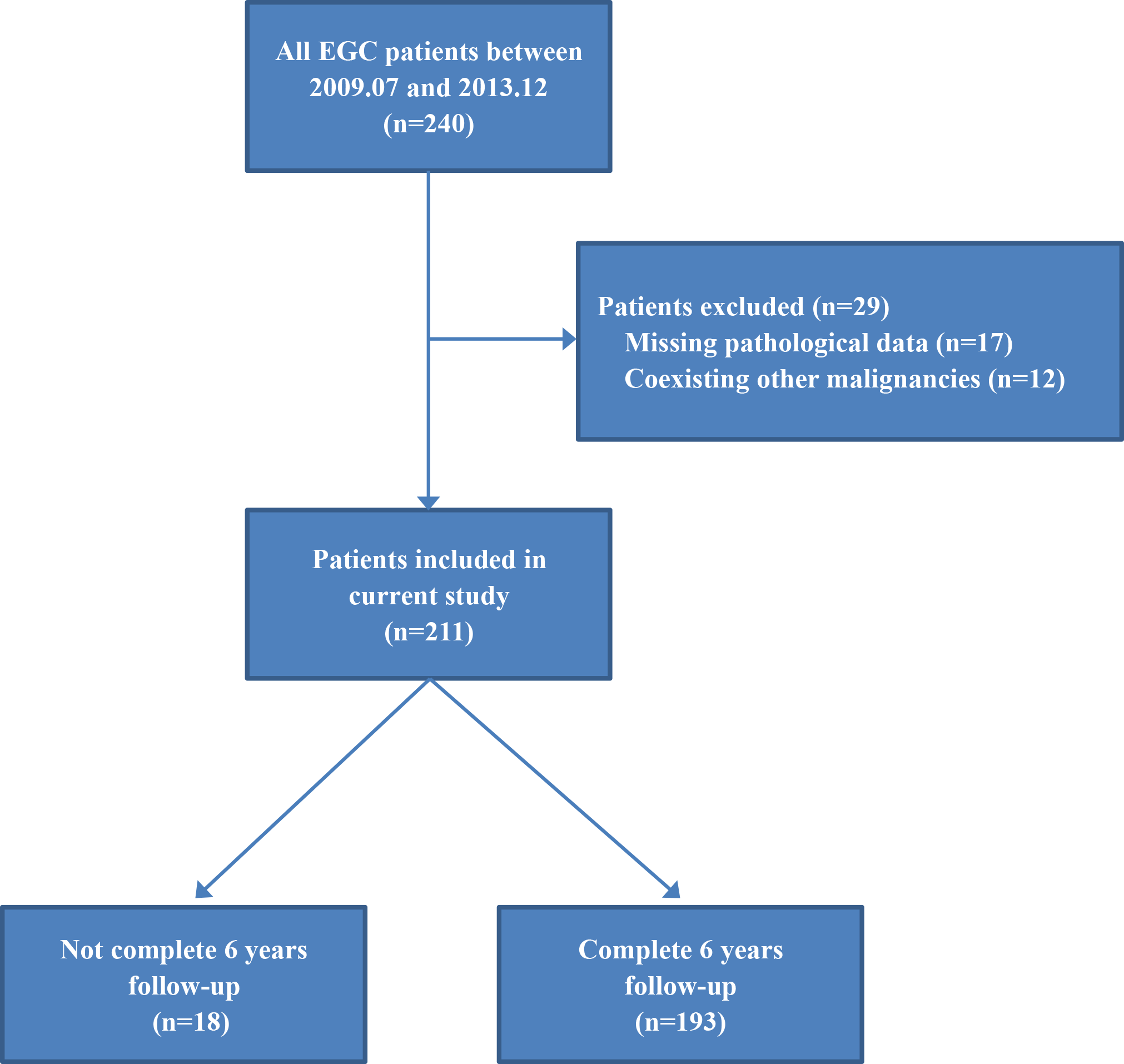

Supplement: Supplementary file 1 — Additional file 1. Flowchart of participants identification. [file 12876_2022_2309_MOESM1_ESM.tif]
