# Supplementary material for: Relationship between HER2 overexpression and long-term outcomes of early gastric cancer: a prospective observational study with a 6-year follow-up
Source: BMC Gastroenterol. 2022 May 13;22:238. doi: 10.1186/s12876-022-02309-7 (PMC9102633; doi:10.1186/s12876-022-02309-7)
Supplement: Supplementary file 2 — Additional file 2. Results of sensitivity analysis by excluding the patients lost to follow-up. [file 12876_2022_2309_MOESM2_ESM.docx]

Table S1. Multivariate analyses for prognostic outcomes excluding patients lost to follow-up (n=193)

| Outcomes | P | Coefficient | SE | Wald | HR (95% CI) |
| --- | --- | --- | --- | --- | --- |
| OS |  |  |  |  |  |
| Age (Per year) | 0.002 | 0.100 | 0.032 | 9.565 | 1.11（1.04-1.18） |
| DSS |  |  |  |  |  |
| Lymphovascular infiltration | 0.005 | 3.423 | 1.225 | 7.811 | 30.66（2.78-338.16） |
| Tumor Recurrence |  |  |  |  |  |
| HER2 overexpression | 0.014 | 1.444 | 0.586 | 6.077 | 4.24（1.34-13.35） |
